# Supplementary material for: Microbial production of multiple short-chain primary amines via retrobiosynthesis
Source: Nat Commun. 2021 Jan 8;12:173. doi: 10.1038/s41467-020-20423-6 (PMC7794544; doi:10.1038/s41467-020-20423-6)
Supplement: Supplementary file 1 — Supplementary Information [file 41467_2020_20423_MOESM1_ESM.pdf]

SUPPLEMENTARY INFORMATION

Microbial production of multiple short-chain primary amines via retrobiosynthesis

Dong In Kim[1,2†], Tong Un Chae[1,2†], Hyun Uk Kim[2,3,4†], Woo Dae Jang[1,2], and Sang Yup Lee[1,2,3*]

[1]Metabolic and Biomolecular Engineering National Research Laboratory, Department of Chemical and Biomolecular Engineering, KAIST Institute for BioCentury, Korea Advanced Institute of Science and Technology (KAIST), Daejeon 34141, Republic of Korea

[2]Systems Metabolic Engineering and Systems Healthcare Cross-Generation Collaborative Laboratory, KAIST, Daejeon 34141, Republic of Korea

[3]KAIST Institute for Artificial Intelligence, BioProcess Engineering Research Center and BioInformatics Research Center, KAIST, Daejeon 34141, Republic of Korea

[4]Systems Biology and Medicine Laboratory, Department of Chemical and Biomolecular Engineering, KAIST, Daejeon 34141, Republic of Korea

[†]These authors contributed equally

*e-mail: leesy@kaist.ac.kr

Supplementary Tables

Supplementary Table 1. Molecular structure and applications of 15 short-chain primary amines (SCPAs) targeted for the microbial production

SCPA	Molecular structure	Applications	Ref
Methylamine	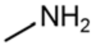	• Precursor of herbicides, pesticides, and insecticides (e.g., metam sodium)<br>• Precursor of Tovex (water gel explosive)<br>• Precursor of solvents (e.g., N-methyl-2-pyrrolidone and methyldiethanolamine)<br>• Precursor of pharmaceuticals	1
Ethylamine	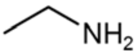	• Food additive<br>• Precursor of herbicides (e.g., atrazine, simazine, ethalfluralin and cycloate)<br>• Precursor of a stabilizer for chlorine in a swimming pool	1, EU Food Improvement Agents[a]
n-Propylamine	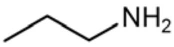	• Food additive<br>• Precursor of agrochemicals and pharmaceuticals	1, EU Food Improvement Agents
iso-Propylamine	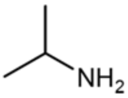	• Food additive<br>• Precursor of herbicides (e.g., bentazon, Roundup, imazapyr, ametryne, atrazine, desmetryn, prometryn, prometon and propazine)<br>• Precursor of nematicides, fungicides, insecticides, pharmaceuticals and surfactants (e.g., fenamiphos and iprodione)	1, EU Food Improvement Agents
n-Butylamine	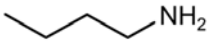	• Food additive<br>• Precursor of rubber accelerators (e.g., 1,3-di-*n*-butylthiourea)<br>• Precursor of plasticizers for nylon 6 resin (*N*-butylbenzenesulfonamide)<br>• Precursor of fungicides (e.g., benomyl)<br>• Precursor of pharmaceuticals (e.g., tolbutamide and antidiabetic drug)	1, EU Food Improvement Agents
iso-Butylamine	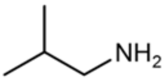	• Food additive	EU Food Improvement Agents
(*R*)-*sec*-Butylamine	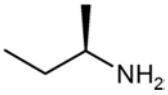	• Food additive<br>• Precursor of herbicides (e.g., bromacil)	1, EU Food Improvement Agents
tert-Butylamine	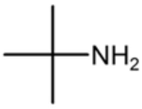	• Precursor of vulcanization accelerators (e.g., N-*tert*-butyl-2-benzothiazylsulfenamide and N-*tert*-butyl-2-benzothiazylsulfenimide)<br>• Precursor of herbicides and insecticides (e.g., terbacil, terbumeton, terbutryn and diafenthiuron)<br>• Precursor of cosmetic stabilizers	1

SCPA	Molecular structure	Applications	Ref
n-Amylamine	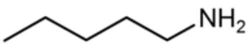	• Food additive<br>• Used together with other isomers (i.e., *iso*-amylamine, *sec*-amylamine and *tert*-amylamine) for drilling application	1, EU Food Improvement Agents
iso-Amylamine	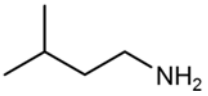	• Precursor of pharmaceuticals and agrochemicals<br>• Used together with other isomers (i.e., *n*-amylamine, *sec*-amylamine and *tert*-amylamine) for drilling application	1
2-Methylbutylamine	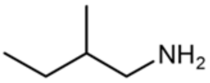	• Food additive	EU Food Improvement Agents
Cyclopentylamine	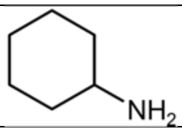	• Precursor of fungicides (e.g., pencycuron)	1
Cyclohexylamine	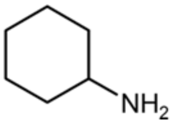	• Corrosion inhibitor<br>• Hardener for epoxy resin, catalyst for polyurethanes and chain terminator for polyamide polymerization<br>• Precursor of vulcanization accelerators (e.g., N-cyclohexyl-2-benzothiazylsulfenamide)<br>• Precursor of plasticizers, emulsifiers and coagulators<br>• Precursor of artificial sweeteners (e.g., cyclamates)<br>• Precursor of herbicides (e.g., hexazinone)	1
Aniline	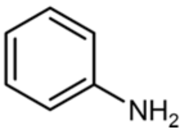	• Vulcanization accelerator<br>• Precursor of antidegradants (e.g., paraphenylenediamines, quinolines and diphenylamine)<br>• Precursor of methylene diphenylene isocyanate, a monomer for polyurethane<br>• Precursor of dyes (e.g., indigo)<br>• Precursor of pesticides and pharmaceuticals	2
Benzylamine	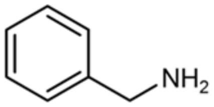	• Corrosion inhibitor<br>• Precursor of pharmaceuticals (e.g., alniditan, lacosamide, moxifloxacin and nebivolol)<br>• Precursor of synthetic textiles	3

[a] EU Food Improvement Agents (https://eur-lex.europa.eu/legal-content/EN/TXT/?uri=CELEX:32012R0872)

Supplementary Table 2. Strains and plasmids used in this study

	Relevant characteristics[a]	Reference or source
**Strains**		
NEB10-beta	*araD*139 Δ(*ara-leu*)7697 *fhuA lacX74 galK* (ϕ80 Δ(*lacZ*)*M15*) *mcrA galU recA1 endA1 nupG rpsL* (Str^R) Δ(*mrr-hsdRMS-mcrBC*)	NEB[b]
BL21(DE3)	F^- *ompT hsdSB* (*rB*$^-$ *mB*$^-$) *gal dcm* (*DE3*)	Invitrogen
W3110	*K12 F*$^-$ (*rmD-rmE*)	CSGC[c]
WL3110	W3110 *ΔlacI*	4
Val	WL3110 *attilvG*::p*tac attilvB*::p*tac* $ilvH^{G41A,\ C50T}$ *ΔilvA ΔpanB ΔleuA*::Cm^R	5
WLGBH	WL3110 *attilvG*::p*tac attilvB*::p*tac* $ilvH^{G41A,\ C50T}$	5
PA01	WL3110 harboring pTac15-vlmD^{opt}	This study
EA01	WL3110 harboring pTac15-vlmD^{opt}-alaD^{opt}	This study
iBA01	Val harboring pKBRilvBNCED and pTac15-vlmD^{opt}	This study
iBA02	WLGBH harboring pKBRilvBNCED and pTac15-vlmD^{opt}-anti-ilvA-leuA-panB	This study
iAA01	Val harboring pKBRilvBNCED and pTac15-vlmD^{opt}-pTac-leuABCD	This study
NC01	WL3110 harboring pTac15-alaD^{opt}	This study
NC02	Val harboring pKBRilvBNCED and pTac15K	This study
NC03	Val harboring pKBRilvBNCED and pTac15-leuABCD	This study
EA01-V94I	WL3110 harboring pTac15-vlmD^{opt}-V94I-alaD^{opt}	This study
EA01-V94L	WL3110 harboring pTac15-vlmD^{opt}-V94L-alaD^{opt}	This study
EA01-V114I	WL3110 harboring pTac15-vlmD^{opt}-V114I-alaD^{opt}	This study
EA01-V114L	WL3110 harboring pTac15-vlmD^{opt}-V114L-alaD^{opt}	This study
EA01-T397A	WL3110 harboring pTac15-vlmD^{opt}-T397A-alaD^{opt}	This study
EA01-T397V	WL3110 harboring pTac15-vlmD^{opt}-T397V-alaD^{opt}	This study
EA01-W399L	WL3110 harboring pTac15-vlmD^{opt}-W399L-alaD^{opt}	This study
EA01-S431T	WL3110 harboring pTac15-vlmD^{opt}-S431T-alaD^{opt}	This study
EA01-S431V	WL3110 harboring pTac15-vlmD^{opt}-S431V-alaD^{opt}	This study

EA01-S431L	WL3110 harboring pTac15-vlmD^{opt}-S431L-alaD^{opt}	This study
EA01-D515K	WL3110 harboring pTac15-vlmD^{opt}-D515K-alaD^{opt}	This study
EA01-D515N	WL3110 harboring pTac15-vlmD^{opt}-D515N-alaD^{opt}	This study
**Plasmids**		
pTac15K	Expression vector, *tac* promoter; p15A ori; Km^{R}	Lab stock
pTrc99A	Expression vector, *trc* promoter; ColE1 ori, Ap^{R}	Pharmacia Biotech
pET-22b(+)	Expression vector, T7 promoter; Ap^{R}	Novagen
pTac15-vlmD^{opt}	pTac15K derivative, containing *E. coli* codon-optimized *vlmD* gene from *S. viridifaciens*	This study
pKBRilvBNCED	Expression vector, containing *ilvBNCED* genes from *E. coli* under *tac* promoter; ColE1 ori; Ap^{R}	5
pTac15-vlmD^{opt}-pTac-leuABCD	pTac15-vlmD^{opt} derivative, containing *leuABCD* genes from *E. coli* under *tac* promoter	This study
pTac15-leuABCD	pTac15-vlmD^{opt}-pTac-leuABCD derivative, containing *leuABCD* genes from *E. coli*	This study
pTac15-vlmD^{opt}-alaD^{opt}	pTac15-vlmD^{opt} derivative, containing *E. coli* codon-optimized *alaD* gene from *G. stearothermophilus*	This study
pTac15-alaD^{opt}	pTac15K derivative, containing *E. coli* codon-optimized *alaD* gene from *G. stearothermophilus*	This study
pET-his$_6$-vlmD^{opt}	pET-22b(+) derivative, containing *E. coli* codon-optimized and N-terminal his$_6$-tagged *vlmD* gene from *S. viridifaciens*	This study
pTac15-vlmD^{opt}-anti-ilvA	pTac15-vlmD^{opt} derivative, containing anti-*ilvA* expression cassette	This study
pTac15-vlmD^{opt}-anti-ilvA-leuA	pTac15-vlmD^{opt}-anti-ilvA derivative, containing anti-*leuA* expression cassette	This study
pTac15-vlmD^{opt}-anti-ilvA-leuA-panB	pTac15-vlmD^{opt}-anti-ilvA-leuA derivative, containing anti-*panB* expression cassette	This study
pTac15-vlmD^{opt}-V94I-alaD^{opt}	pTac15-vlmD^{opt}-alaD^{opt} derivative, containing V94I variant of *E. coli* codon-optimized *vlmD* gene from *S. viridifaciens*	This study
pTac15-vlmD^{opt}-V94L-alaD^{opt}	pTac15-vlmD^{opt}-alaD^{opt} derivative, containing V94L variant of *E. coli* codon-optimized *vlmD* gene from *S. viridifaciens*	This study
pTac15-vlmD^{opt}-V114I-alaD^{opt}	pTac15-vlmD^{opt}-alaD^{opt} derivative, containing V114I variant of *E. coli* codon-optimized *vlmD* gene from *S. viridifaciens*	This study
pTac15-vlmD^{opt}-V114L-alaD^{opt}	pTac15-vlmD^{opt}-alaD^{opt} derivative, containing V114L variant of *E. coli* codon-optimized *vlmD* gene from *S. viridifaciens*	This study
pTac15-vlmD^{opt}-T397A-alaD^{opt}	pTac15-vlmD^{opt}-alaD^{opt} derivative, containing T397A variant of *E. coli* codon-optimized *vlmD* gene from *S. viridifaciens*	This study
pTac15-vlmD^{opt}-T397V-alaD^{opt}	pTac15-vlmD^{opt}-alaD^{opt} derivative, containing T397V variant of *E. coli* codon-optimized *vlmD* gene from *S. viridifaciens*	This study
pTac15-vlmD^{opt}-W399L-alaD^{opt}	pTac15-vlmD^{opt}-alaD^{opt} derivative, containing W399L variant of *E. coli* codon-optimized *vlmD* gene from *S. viridifaciens*	This study
pTac15-vlmD^{opt}-S431T-alaD^{opt}	pTac15-vlmD^{opt}-alaD^{opt} derivative, containing S431T variant of *E. coli* codon-optimized *vlmD* gene from *S. viridifaciens*	This study

pTac15-vlmD^{opt}-S431V-alaD^{opt}	pTac15-vlmD^{opt}-alaD^{opt} derivative, containing S431V variant of *E. coli* codon-optimized *vlmD* gene from *S. viridifaciens*	This study
pTac15-vlmD^{opt}-S431L-alaD^{opt}	pTac15-vlmD^{opt}-alaD^{opt} derivative, containing S431L variant of *E. coli* codon-optimized *vlmD* gene from *S. viridifaciens*	This study
pTac15-vlmD^{opt}-D515K-alaD^{opt}	pTac15-vlmD^{opt}-alaD^{opt} derivative, containing D515K variant of *E. coli* codon-optimized *vlmD* gene from *S. viridifaciens*	This study
pTac15-vlmD^{opt}-D515N-alaD^{opt}	pTac15-vlmD^{opt}-alaD^{opt} derivative, containing D515N variant of *E. coli* codon-optimized *vlmD* gene from *S. viridifaciens*	This study

[a] Abbreviations: Ap, ampicillin; Cm, chloramphenicol; Km, kanamycin; Str, strptomycine; ^{R}, resistance.

[b] NEB, New England Biolabs, Ipswich, MA

[c] CSGC, Coli Genetic Stock Center, New Haven, CT

Supplementary Table 3. Primers used in this study

Name	Sequence (5'→3')
vlmD_F1	AGACAGGAATTCATGAGTACCAGCTCTGCCAGTTC
vlmD_R1	AGACAGGGTACCTTAGCTGCCACCGCCATC
vlmD_F2	AGACAGCATATGCATCATCACCATCACCACAGTACCAGCTCTGCCAGTTC C
vlmD_R2	AGACAGGAATTCTTAGCTGCCACCGCCATC
alaD_F1	AGACAGGGTACCTTTCACACAGGAAACAATGAAAATTGGTATACCGAAG GAA
alaD_R1	AGACAGCTGCAGTCATCCTTGCAGAAGAGAATGG
alaD_F2	AGACAGGAATTCATGAAAATTGGTATACCGAAGGAA
alaD_R2	AGACAGGAATTCTCATCCTTGCAGAAGAGAATGG
leuABCD_F1	GCTCGTATAATGTGTGGAATTGTGAGCGGATAACAATTTCACACAGGAAA CA ATGAGCCAGCAAGTCATTATTTT
leuABCD_R1	TTAATTCATAAACGCAGGTTGTTT
leuABCD_F2	AGACAGCTGCAGGCTGTTGACAATTAATCATCGGCTCGTATAATGTGTGG AATTGTG
leuABCD_R2	AGACAGCTGCAGTTAATTCATAAACGCAGGTTGTTT
inv_F1	ATGAGCCAGCAAGTCATTATTTTC
inv_R1	GAATTCTGTTTCCTGTGTGAAATTG
inv_F2	CAGCAACCGCACCTGTGG
inv_R2	GCGCCTATATCGCCGACAT
anti-ilvA_F	GCCCGATCTTCCCCATCGGTGATGTCGGCGATATAGGCGCTAACACCGTGC GTGTTGACTATT
anti-ilvA_R	ATCGTGGCCGGCATCACCGGCGCCACAGGTGCGGTTGCTGTATAAACGCA GAAAGGCCCAC
inv_F3	CAGGACCACTTCTGCGCTC
inv_R3	CAACTTTATCCGCCTCCATCC
anti-leuA_F	CGGCAACAATTAATAGACTGGATGGAGGCGGATAAAGTTGTAACACCGTG CGTGTTGACTATT
anti-leuA_R	AACCAGCCAGCCGGAAGGGCCGAGCGCAGAAGTGGTCCTGTATAAACGC AGAAAGGCCCAC
inv_F4	AAAGTTCTGCTATGTGGCGC
inv_R4	AAAAGTGCTCATCATTGGAAAAC
anti-panB_F	TTTTCGCCCCGAAGAACGTTTTCCAATGATGAGCACTTTTTAACACCGTG CGTGTTGACTATT
anti-panB_R	CGTCAACACGGGATAATACCGCGCCACATAGCAGAACTTTTATAAACGCA GAAAGGCCCAC
V94I_F	ATTACGAGCGATCTGGACTATCAGC
V94L_F	CTGACGAGCGATCTGGACTATCAGC
V94_R	TTGATAGCCTGCGAAGTTGC
V114I_F	ATTGGTGATCCGTATGAAAGCTCTAGTT
V114L_F	CTGGGTGATCCGTATGAAAGCTCTAGTT

V114_R	ATTGTTCAGATGACGATTCAGGTAG
T397A_F	GCGCCGTGGGCCTGTGGTG
T397V_F	GTGCCGTGGGCCTGTGGTG
T397_R	ACCCATCCATTTGTGGCC
W399L_F	CTGGCCTGTGGTGTGTACATGACC
W399_R	CGGCGTACCCATCCATTT
S431T_F	ACCCGCAACGGTTTCTCCTCAC
S431V_F	GTGCGCAACGGTTTCTCCTCAC
S431L_F	CTGCGCAACGGTTTCTCCTCAC
S431_R	GCCGGCAAACGTGGTG
D515K_F	AAAAACGAACAACGCACGTATGTG
D515N_F	AACAACGAACAACGCACGTATGTG
D515_R	TTCATACACGGTTTCACAGCTC

Supplementary Table 4. DNA sequences of codon-optimized *Streptomyces viridifaciens vlmD* and *Geobacillus stearothermophilus alaD* genes

Gene	Codon-optimized sequence
vlmD	ATGAGTACCAGCTCTGCCAGTTCCGGTCCGGACCTGCCGTTTGGTCCGGAAG<br>ATACGCCGTGGCAAAAAGCTTTCTCCCGTCTGCGTGCCGTTGATGGCGTCCCG<br>CGTGTGACCGCCCCGTCATCGGACCCGCGTGAAGTTTACATGGATATTCCGG<br>AAATCCCGTTTAGCAAAGTCCAGATTCCGCCGGACGGTATGGATGAACAGCA<br>ATACGCAGAAGCTGAATCTCTGTTTCGTCGCTATGTGGATGCCCAGACCCGCA<br>ACTTCGCAGGCTATCAAGTTACGAGCGATCTGGACTATCAGCATCTGTCTCAC<br>TACCTGAATCGTCATCTGAACAATGTTGGTGATCCGTATGAAAGCTCTAGTTA<br>CACCCTGAACAGCAAAGTTCTGGAACGCGCCGTCCTGGATTATTTTGCCAGTC<br>TGTGGAATGCAAAATGGCCGCACGACGCATCCGATCCGGAAACCTATTGGGG<br>CTACGTGCTGACGATGGGTTCCTCAGAAGGCAACCTGTATGGTCTGTGGAAT<br>GCCCGCGACTACCTGAGCGGTAAACTGCTGCGTCGCCAACATCGTGAAGCTG<br>GCGGTGATAAAGCGTCAGTGGTTTATACCCAGGCACTGCGTCATGAAGGCCA<br>ATCGCCGCACGCCTACGAACCGGTTGCATTTTTCAGTCAGGATACCCATTATT<br>CCCTGACGAAAGCGGTCCGCGTGCTGGGCATTGACACCTTTCACTCTATCGGT<br>TCGAGCCGTTATCCGGATGAAAACCCGCTGGGTCCGGGCACCCCGTGGCCGA<br>CGGAAGTTCCGAGCGTCGATGGCGCTATTGATGTTGACAAACTGGCGTCACT<br>GGTCCGCTTTTTCGCCTCGAAAGGTTATCCGATCCTGGTTAGCCTGAATTACG<br>GCTCTACCTTCAAGGGTGCGTATGATGATGTGCCGGCGGTTGCACAGGCAGT<br>CCGTGATATTTGCACGGAATACGGCCTGGACCGTCGCCGTGTGTATCATGATC<br>GCTCTAAAGATAGTGACTTTGATGAACGTAGTGGTTTCTGGATTCACATTGAT<br>GCCGCACTGGGTGCTGGTTATGCCCCGTATCTGCAAATGGCCCGCGACGCAG<br>GCATGGTCGAAGAAGCACCGCCGGTGTTTGATTTCCGTCTGCCGGAAGTTCAT<br>TCTCTGACCATGAGTGGCCACAAATGGATGGGTACGCCGTGGGCCTGTGGTG<br>TGTACATGACCCGTACCGGTCTGCAAATGACCCCGCCGAAATCTAGTGAATA<br>TATCGGTGCAGCTGACACCACGTTTGCCGGCAGCCGCAACGGTTTCTCCTCAC<br>TGCTGCTGTGGGATTATCTGTCCCGTCATTCATACGATGACCTGGTTCGCCTG<br>GCGGCCGACTGTGATCGTCTGGCTGGCTATGCGCACGATCGCCTGCTGACCCT<br>GCAAGACAAACTGGGTATGGATCTGTGGGTGGCACGTTCGCCGCAAAGCCTG<br>ACGGTTCGCTTTCGTCAGCCGTGCGCTGACATTGTCCGTAAATACTCGCTGAG<br>CTGTGAAACCGTGTATGAAGATAACGAACAACGCACGTATGTGCATCTGTAC<br>GCGGTTCCGCACCTGACCCGTGAACTGGTCGACGAACTGGTTCGTGATCTGC<br>GTCAGCCGGGTGCCTTCACCAATGCTGGCGCGCTGGAAGGTGAAGCATGGGC<br>AGGCGTGATTGATGCGCTGGGTCGTCCGGACCCGGATGGCACCTATGCTGGT<br>GCGCTGTCCGCACCGGCATCAGGTCCGCGTAGCGAAGATGGCGGTGGCAGCT<br>AA
alaD	ATGAAAATTGGTATACCGAAGGAAATTAAAAATAATGAAAACCGCGTTGCCA<br>TTACACCTGCCGGTGTGATGACTTTAGTCAAGGCCGGGCACGACGTGTATGTC<br>GAAACCGAAGCAGGGGCGGGCAGCGGCTTTTCCGATTCGGAATATGAAAAA<br>GCAGGAGCAGTTATAGTTACTAAGGCCGAAGACGCCTGGGCGGCTGAAATGG<br>TTTTGAAAGTAAAGGAGCCCCTGGCAGAGGAATTTCGGTACTTTAGACCGGG<br>ACTTATATTATTTACCTACCTTCATTTAGCGGCGGCCGAGGCGTTGACGAAGG<br>CCCTGGTGGAACAGAAAGTTGTAGGTATCGCCTACGAGACGGTCCAACTGGC<br>CAACGGAAGCCTTCCGTTATTAACCCCTATGTCCGAAGTGGCCGGGCGGATG<br>TCCGTCCAGGTCGGAGCACAGTTTTTAGAAAAGCCACACGGTGGGAAGGGCA<br>TTTTATTGGGAGGGGTTCCGGGGGTACGGCGTGGAAAAGTTACGATTATTGG<br>GGGGGGAACTGCTGGCACTAACGCTGCCAAGATAGCGGTGGGGTTGGGGGCT<br>GACGTCACGATACTTGATATAAACGCGGAACGCCTGAGAGAGTTAGACGATT<br>TGTTCGGAGATCAAGTTACCACGTTGATGAGCAATTCATATCACATTGCAGA

	ATGCGTTAGAGAAAGTGACCTGGTAGTGGGTGCTGTGCTGATCCCCGGGGCA<br>AAGGCCCCAAAGTTAGTAACCGAGGAGATGGTCCGTAGCATGACGCCAGGTA<br>GTGTCTTAGTCGATGTCGCCATTGACCAGGGTGGAATCTTCGAAACAACCGA<br>CCGTGTCACGACCCATGACGACCCCACCTATGTCAAACACGGTGTCGTACAC<br>TATGCGGTCGCGAACATGCCAGGAGCTGTACCTCGCACCAGCACGTTTGCGC<br>TTACCAATGTGACCATCCCGTATGCCTTGCAGATCGCCAACAAGGGATACCG<br>TGCTGCCTGTTTAGATAATCCAGCGTTGTTAAAGGGTATCAACACCTTGGATG<br>GACATATCGTTTACGAGGCCGTTGCCGCAGCACATAATATGCCATATACCGA<br>TGTCCATTCTCTTCTGCAAGGATGA

Supplementary Figures

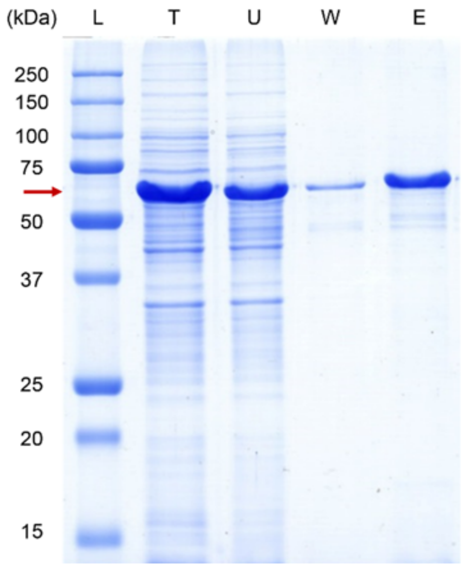

Supplementary Fig. 1. SDS-PAGE analysis of the purified His_6-tagged VlmD. The lanes are: L, molecular weight markers; T, total proteins; U, unbound proteins; W, washed proteins with 7.5 mM of imidazole; and E, eluted proteins with 150 mM of imidazole. Red arrow indicates the expected molecular weight of His_6-tagged VlmD (66.9 kDa). Experiment was conducted once.

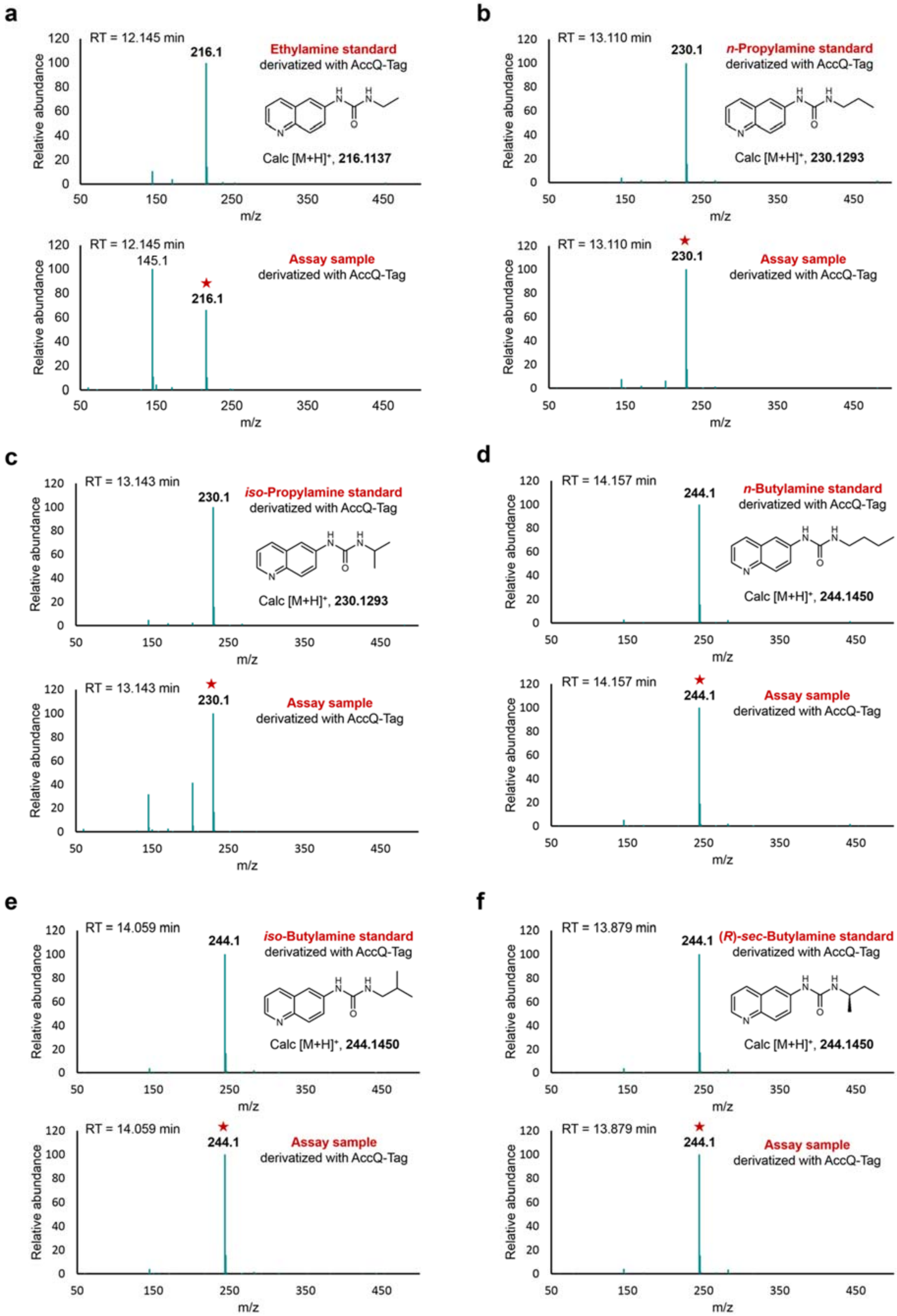
a
RT = 12.145 min
216.1
Ethylamine standard
derivatized with AccQ-Tag
Calc [M+H]+, 216.1137
RT = 12.145 min
145.1
216.1
Assay sample
derivatized with AccQ-Tag
b
RT = 13.110 min
230.1
n-Propylamine standard
derivatized with AccQ-Tag
Calc [M+H]+, 230.1293
RT = 13.110 min
230.1
Assay sample
derivatized with AccQ-Tag
c
RT = 13.143 min
230.1
iso-Propylamine standard
derivatized with AccQ-Tag
Calc [M+H]+, 230.1293
RT = 13.143 min
230.1
Assay sample
derivatized with AccQ-Tag
d
RT = 14.157 min
244.1
n-Butylamine standard
derivatized with AccQ-Tag
Calc [M+H]+, 244.1450
RT = 14.157 min
244.1
Assay sample
derivatized with AccQ-Tag
e
RT = 14.059 min
244.1
iso-Butylamine standard
derivatized with AccQ-Tag
Calc [M+H]+, 244.1450
RT = 14.059 min
244.1
Assay sample
derivatized with AccQ-Tag
f
RT = 13.879 min
244.1
(R)-sec-Butylamine standard
derivatized with AccQ-Tag
Calc [M+H]+, 244.1450
RT = 13.879 min
244.1
Assay sample
derivatized with AccQ-Tag
Relative abundance
m/z

g

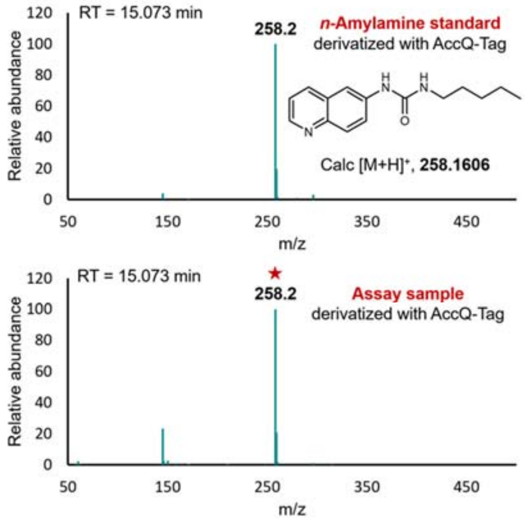
RT = 15.073 min
258.2
n-Amylamine standard
derivatized with AccQ-Tag
Calc [M+H]+, 258.1606
RT = 15.073 min
258.2
Assay sample
derivatized with AccQ-Tag
Relative abundance
m/z

h

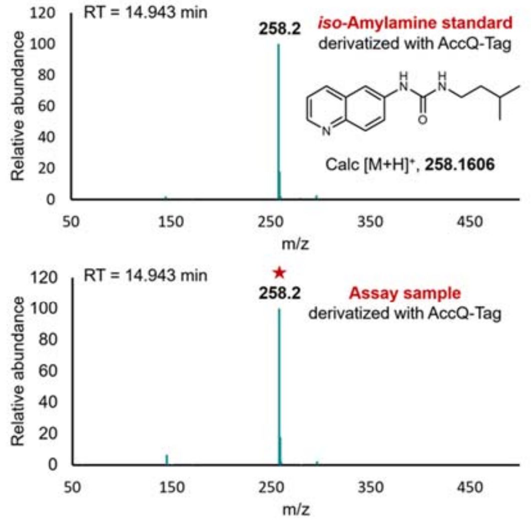
RT = 14.943 min
258.2
iso-Amylamine standard
derivatized with AccQ-Tag
Calc [M+H]+, 258.1606
RT = 14.943 min
258.2
Assay sample
derivatized with AccQ-Tag
Relative abundance
m/z

i

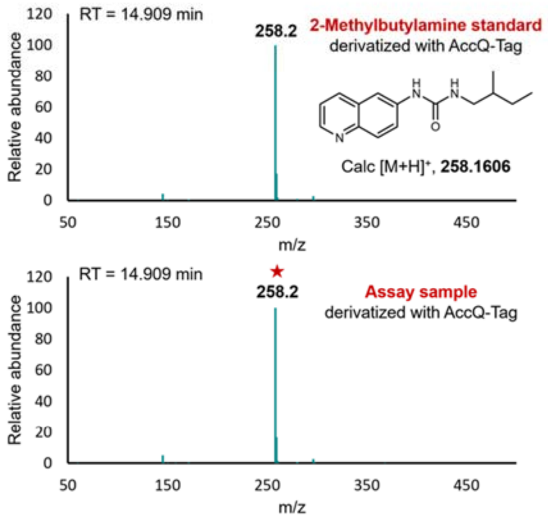
RT = 14.909 min
258.2
2-Methylbutylamine standard
derivatized with AccQ-Tag
Calc [M+H]+, 258.1606
RT = 14.909 min
258.2
Assay sample
derivatized with AccQ-Tag
Relative abundance
m/z

j

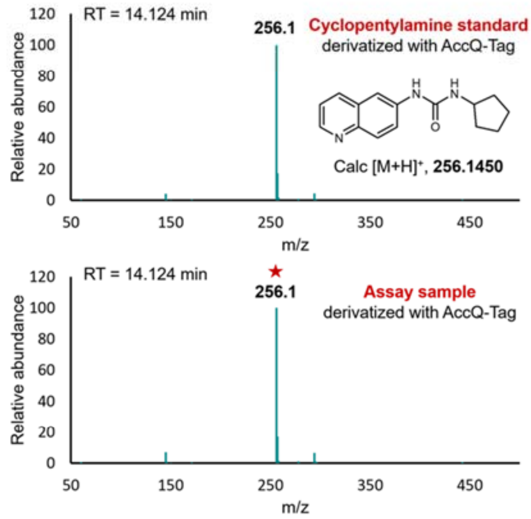
RT = 14.124 min
256.1
Cyclopentylamine standard
derivatized with AccQ-Tag
Calc [M+H]+, 256.1450
RT = 14.124 min
256.1
Assay sample
derivatized with AccQ-Tag
Relative abundance
m/z

k

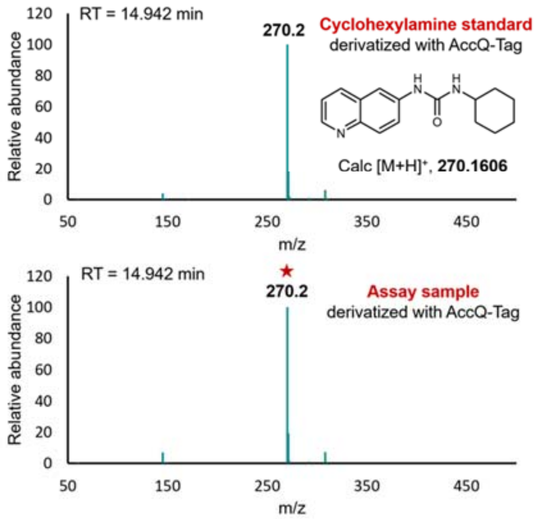
RT = 14.942 min
270.2
Cyclohexylamine standard
derivatized with AccQ-Tag
Calc [M+H]+, 270.1606
RT = 14.942 min
270.2
Assay sample
derivatized with AccQ-Tag
Relative abundance
m/z

l

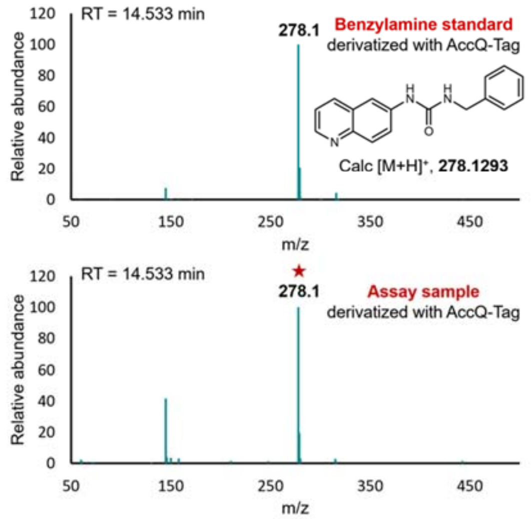
RT = 14.533 min
278.1
Benzylamine standard
derivatized with AccQ-Tag
Calc [M+H]+, 278.1293
RT = 14.533 min
278.1
Assay sample
derivatized with AccQ-Tag
Relative abundance
m/z

Supplementary Fig. 2. LC-MS profiles of the short-chain primary amine (SCPA) standards and in vitro enzyme assay samples derivatized with AccQ-Tag. The ion spectra of the standards and in vitro enzyme assay samples derivatized with AccQ-Tag for the following SCPAs from their corresponding precursors: **a** ethylamine from L-alanine; **b** *n*-propylamine from L-2-aminobutyrate; **c** *iso*-propylamine from 2-aminoisobutyrate; **d** *n*-butylamine from L-norvaline; **e** *iso*-butylamine from L-valine; **f** (*R*)-*sec*-butylamine from L-isovaline; **g** *n*-amylamine from L-norleucine; **h** *iso*-amylamine from L-leucine; **i** 2-methylbutylamine from L-isoleucine; **j** cyclopentylamine from 1-aminocyclopentanecarboxylic acid; **k** cyclohexylamine from 1-aminocyclohexanecarboxylic acid; and **l** benzylamine from L-2-phenylglycine. Experiments were conducted in triplicates, and the representative mass spectra are shown.

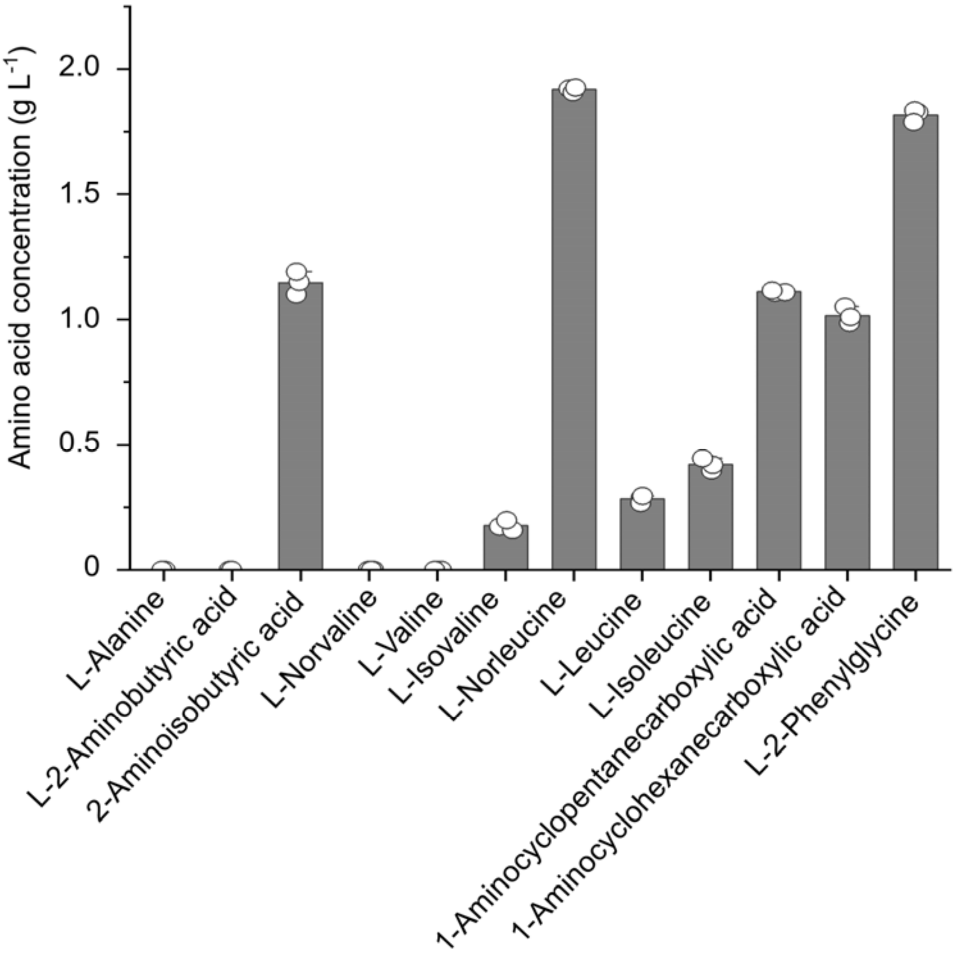

Supplementary Fig. 3. The concentrations of amino acid precursors of the 12 short-chain primary amine (SCPAs) from flask cultivation of the PA01 strain. Flask cultures for the in vivo production of the 12 SCPAs were conducted with supplementation of 2 g L^{-1} of the corresponding amino acid precursors. Experiments were conducted in triplicates. Data are presented as mean values ± s.d. Source data are provided as a Source Data file.

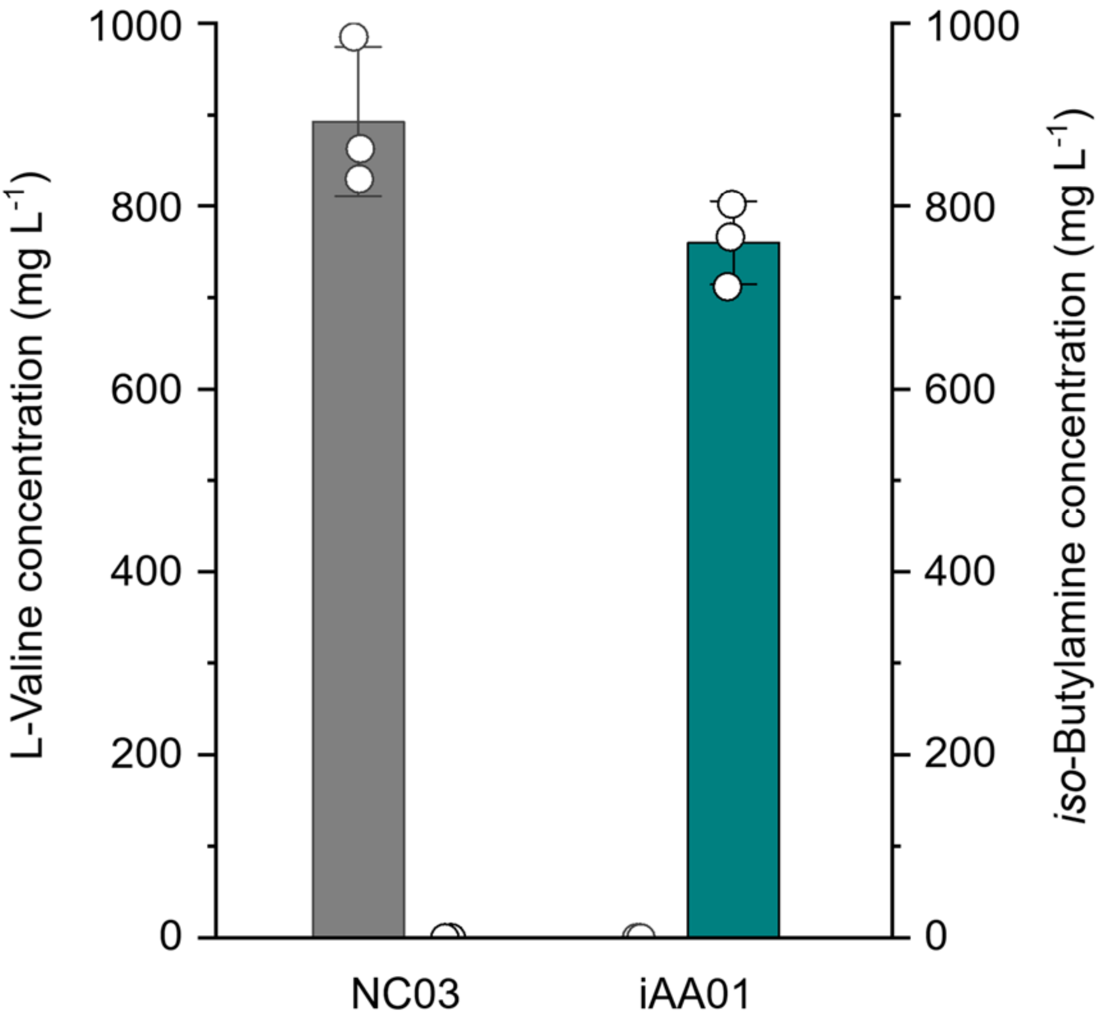

Supplementary Fig. 4. The concentrations of L-valine and *iso*-butylamine produced by flask cultures of the NC03 and iAA01 strains, respectively. Experiments were conducted in triplicates. Data are presented as mean values ± s.d. Source data are provided as a Source Data file.

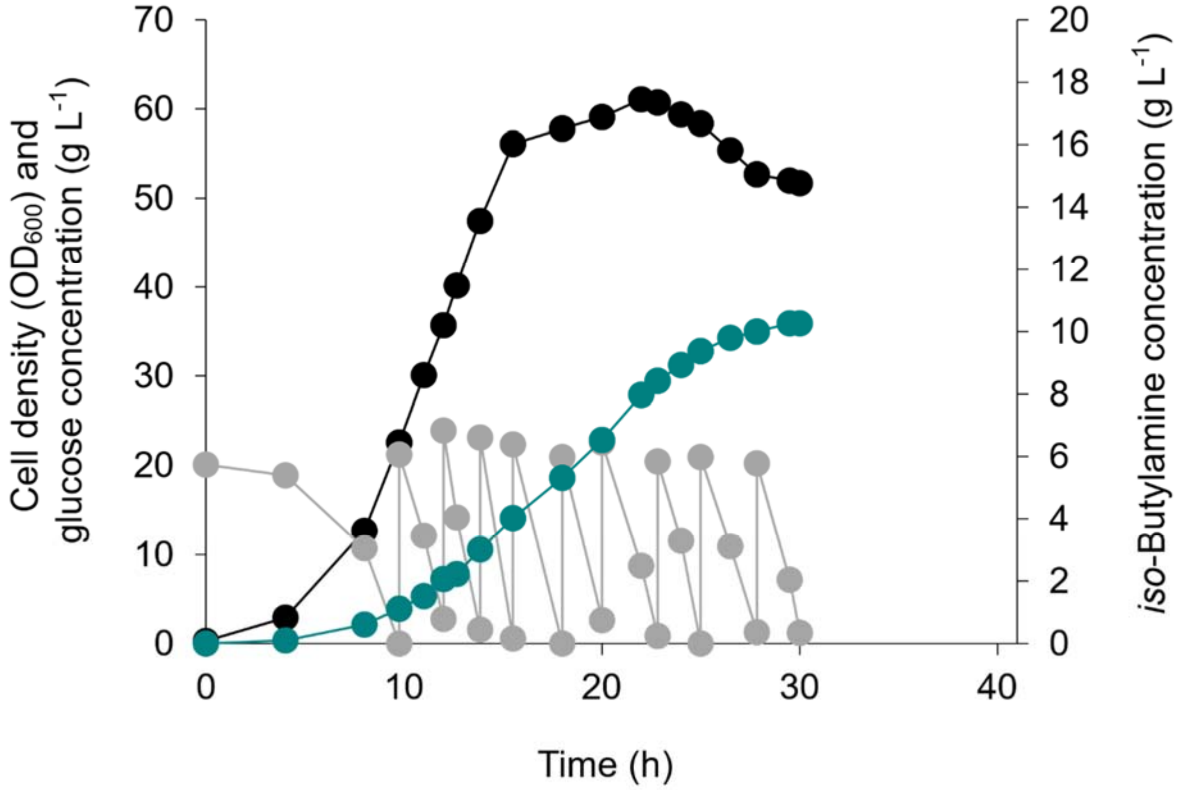

Supplementary Fig. 5. Fed-batch fermentation profile of the iBA02 strain performed independently. Cell density (OD_{600}), glucose concentration, and *iso*-butylamine concentration are presented with black, gray and green circles, respectively. Source data are provided as a Source Data file.

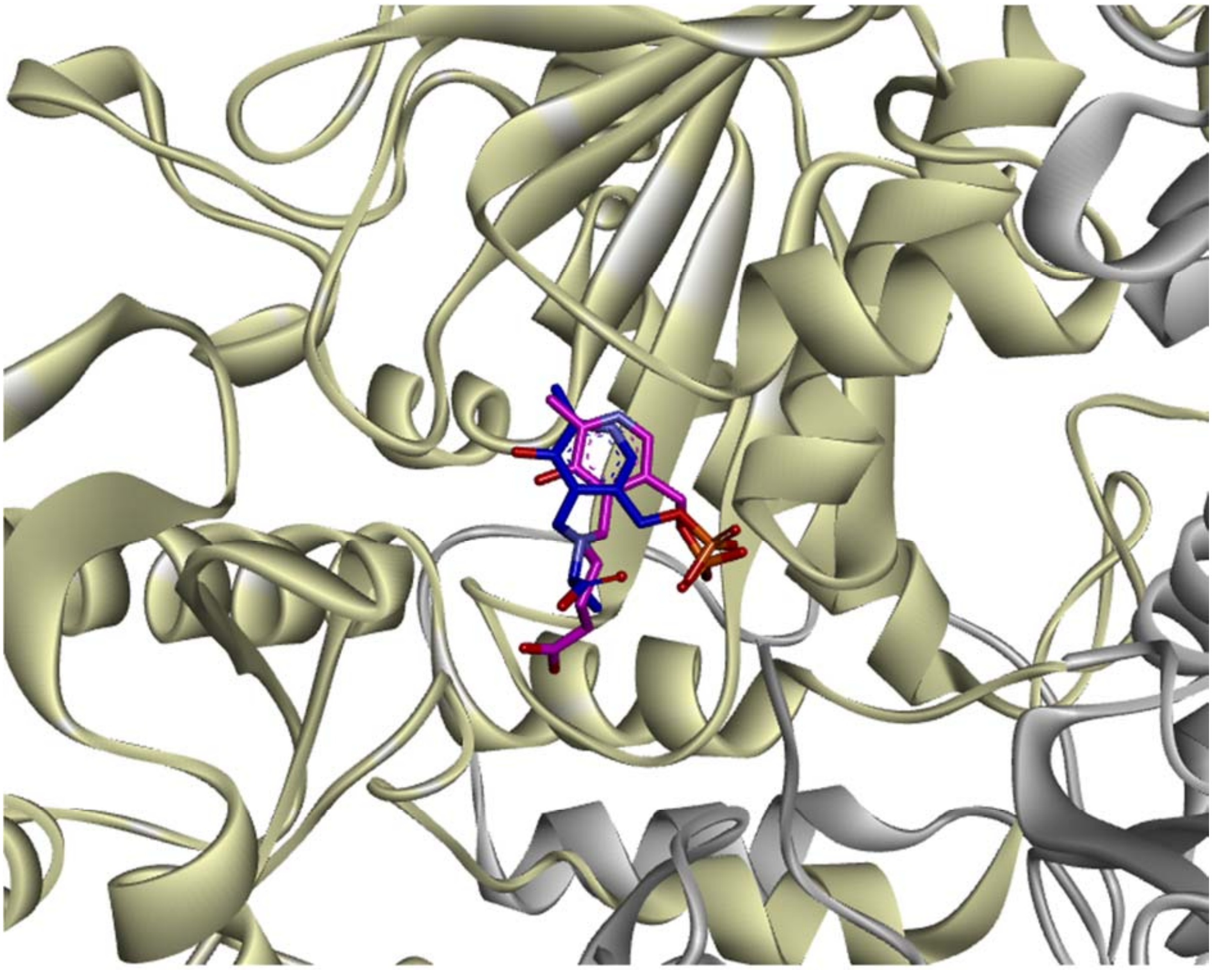

Supplementary Fig. 6. Binding mode of PLP-L-alanine in the homology model of VlmD in comparison with the co-crystal structure of GAD67 bound with PLP-GABA. Similar binding modes were observed for the two dockings on the basis of the superimposition between the homology model of VlmD and the co-crystal structure of GAD67 (PDB ID: 2OKJ). The PLP-L-alanine and PLP-GABA molecules are presented in blue and purple, respectively.

Supplementary References

1. Peter, R., Karsten, E., Erhard, H., Roland, R. & Hartmut, H. in *Amines, Aliphatic* in *Ullmann's Encylopedia of Industrial Chemistry* (ed. Barbara E.) 1-50 (Wiley-VCH, Weinheim, 2015).
2. Thomas, K. et al. in *Aniline* in *Ullmann's Encyclopedia of Industrial Chemistry* (ed. Barbara E.) 465-477 (Wiley-VCH, Weinheim, 2011).
3. Heuer, L. in *Benzylamine* in *Ullmann's Encyclopedia of Industrial Chemistry* (ed. Barbara E.) 385-387 (Wiley-VCH, Weinheim, 2006).
4. Lee, K.H., Park, J.H., Kim, T.Y., Kim, H.U. & Lee, S.Y. Systems metabolic engineering of *Escherichia coli* for L-threonine production. *Mol. Syst. Biol.* **3**, 149 (2007).
5. Park, J.H., Lee, K.H., Kim, T.Y. & Lee, S.Y. Metabolic engineering of *Escherichia coli* for the production of L-valine based on transcriptome analysis and *in silico* gene knockout simulation. *Proc. Natl. Acad. Sci. USA* **104**, 7797-7802 (2007).
