# Supplementary material for: Microbial production of multiple short-chain primary amines via retrobiosynthesis
Source: Nat Commun. 2021 Jan 8;12:173. doi: 10.1038/s41467-020-20423-6 (PMC7794544; doi:10.1038/s41467-020-20423-6)
Supplement: Supplementary file 3 — Descriptions of Additional Supplementary Files [file 41467_2020_20423_MOESM3_ESM.docx]

**Descriptions of Additional Supplementary Files**

**Supplementary Dataset 1:**

Reaction rules used for the retrobiosynthesis in this study

**Supplementary Dataset 2:**

Precursors predicted for the 15 short-chain primary amines
